# Effect of Oscillating Magnetic Fields (OMFs) and Pulsed Electric Fields (PEFs) on Supercooling Preservation of Atlantic Salmon (*Salmo salar* L.) Fillets

**DOI:** 10.3390/foods13162525

**Published:** 2024-08-13

**Authors:** Dongyoung Lee, Jinwen Tang, Seung Hyun Lee, Soojin Jun

**Affiliations:** 1 Department of Molecular Biosciences and Bioengineering, University of Hawaii at Manoa, Honolulu, HI 96822, USA; lee272@hawaii.edu; 2 Department of Human Nutrition, Food and Animal Sciences, University of Hawaii at Manoa, Honolulu, HI 96822, USA; tang9@hawaii.edu; 3 Department of Biosystems Machinery Engineering, Chungnam National University, Daejeon 34134, Republic of Korea

**Keywords:** supercooling, oscillating magnetic field, pulsed electric field, salmon, quality

## Abstract

Salmon, rich in protein and omega-3 fatty acids, has a short shelf life of 1 to 3 days when stored at 2 to 8 °C. Freezing, used for long-term preservation, often results in ice crystal formation. Ice crystals can cause structural damage, leading to cell wall rupture, which can affect the texture and cause nutrient loss. Ultimately, this process reduces the overall quality of the salmon. Supercooling, which cools food below its freezing temperature without forming ice crystals, offers an alternative. This study investigated the effects of oscillating magnetic fields (OMFs) and pulsed electric fields (PEFs) on ice crystal formation during salmon supercooling. The results showed that using OMFs and PEFs in supercooling reduced the storage temperature of salmon, maintaining a similar thiobarbituric acid reactive substances (TBARS) value to that of frozen and refrigerated samples. There was no significant difference in meat color between the fresh and frozen samples, and drip loss weight was comparable between the fresh and supercooled samples. The microbiological counts were the lowest in the supercooled samples compared to the frozen and refrigerated ones. These findings suggest that supercooling storage with OMFs and PEFs can mitigate quality degradation in salmon typically associated with freezing.

## 1. Introduction

Salmon is a highly valued commercial seafood, known for its rich omega-3 fatty acids and vitamin D content, contributing to its popularity among consumers. The demand for salmon has increased significantly, with consumption now three times higher than in 1980, driven by its nutritional benefits and delicious taste [1]. Once considered a luxury food, salmon has become one of the most popular fish. This surge in demand has led to rapid growth in the global salmon trade, with aquaculture now accounting for 70% of the market, reaching 2.5 million tons annually [2]. However, preserving salmon, especially in ready-to-eat forms, presents significant challenges. Traditional preservation methods such as freezing or chilling by ice can cause structural damage to the muscle tissues of salmon, affecting its texture and flavor [3].

Innovations in preservation technology are vital for improving the quality and safety of salmon fillets. Modified atmosphere packaging (MAP) has been shown to extend the shelf life of salmon by reducing microbial growth and maintaining sensory qualities [4]. However, MAP has disadvantages, such as the potential for anaerobic bacteria growth due to reduced oxygen levels, which can lead to spoilage and food safety concerns [5]. Superchilling is another promising technology that maintains the temperature of salmon just below the freezing point, thus slowing down microbial growth and extending shelf life [6]. Despite its advantages, superchilling can also lead to issues such as partial freezing, which may cause textural changes and drip loss upon thawing [7,8].

To address these limitations, supercooling has emerged as a new food processing technology that can decrease the temperature of food products below the freezing point without forming ice crystals. With this technology, food can be stored at low temperatures for longer without the risk of causing ice crystal damage [9]. However, the supercooling state in food is not easy to achieve, and it is even more challenging to maintain for a long enough time as supercooling food is theoretically unstable because of thermodynamics, food properties, and stochastic ice nucleation that may occur during the supercooling process [10]. Recently, techniques in electric and magnetic fields have been developed to control the nucleation phenomenon [11].

In recent years, some studies have investigated the use of an oscillating magnetic field (OMF) and pulsed electric field (PEF) to prevent ice nucleation during freezing [12]. The hypothesis is that electric and magnetic fields have potential interference and rotation effects on water molecules in food near freezing temperatures. The combined treatment could increase the mobility of water molecules [13] and ultimately prevent the aggregation of water molecules. According to current research, due to the diamagnetism of water, the oscillating magnetic field may cause water molecules to vibrate, thereby preventing the formation of ice nuclei under freezing temperatures [14]. Also, the electric field could redirect and rearrange water molecules because of electric dipoles in water molecules [15]. These observations suggest that OMFs and PEFs may prevent ice nucleation during freezing. Indeed, it was demonstrated that a combination of an OMF and PEF suppressed ice formation within a chicken breast and maintained a supercooling state of −7 °C [16]. Kang et al. [17] showed that a PEF combined with an OMF inhibited ice nucleation, storing tuna fillets in a supercooling state of −3.2 °C for eight days. A study on beef steak also showed that supercooling storage could extend the shelf life of beef while maintaining its freshness [18]. However, several engineering perspectives on the application of supercooling in food storage, including the impacts on food safety and its applicability when using this technology in large-scale production, still need to be evaluated. This experiment investigated the quality of fresh salmon after 10 days of supercooling storage under different conditions. By comparing the results with those of salmon samples that were kept in refrigerated and frozen environments for 10 days, the quality of the supercooled salmon fillets was assessed based on various factors.

## 2. Materials and Methods

### 2.1. Sample Preparation

Atlantic salmon fillets (farm-raised) were filleted and packaged by a retail market in Honolulu, HI, USA. All fillets were labeled to be used or frozen within four days. Each package was transferred to the laboratory under controlled temperature conditions using a cooling box maintained at 2 to 4 °C on the same day. Three packs of fillets were randomly purchased for each experimental group, and one sample was taken from each fillet pack. The top loin parts of the fillets were cut into pieces with dimensions of 12 × 6 × 3 cm, weighing 150.03 g ± 9.12 g. The samples were wrapped with polyethylene plastic to prevent moisture loss during the experiment. On the day of purchase, all samples were stored in three different temperature conditions. Samples were refrigerated at 4 °C, supercooled at −3 °C, and frozen at −18 °C during storage. Each storage experiment was repeated three times. Quality assessments were performed on the differently treated samples after 10 days of storage. All appliances and the operating environment were sterilized.

### 2.2. Supercooling Preservation

**Figure 1:**
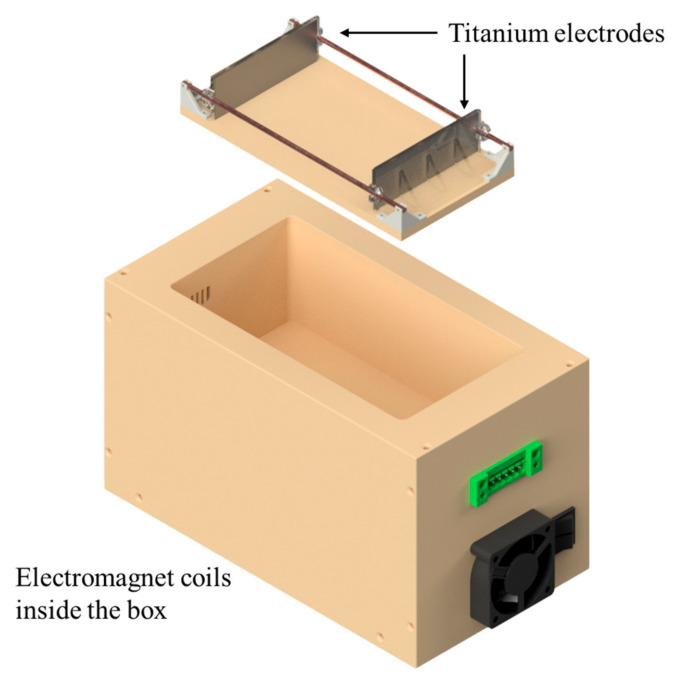
The supercooling chamber has electromagnet coils for the OMF and titanium electrodes for the PEF. The sample was located between a pair of electrodes.

The chamber for the customized supercooling system designed to generate an OMF (oscillating magnetic field) and a PEF (pulsed electric field) was placed in a 7.1 cu. ft chest freezer, as shown in Figure 1. A detailed device setup was also described in our previous work [19]. The OMF signals were generated using a power supply based on an Insulated-gate bipolar transistor (IGBT). The PEF signal was set to 20 kHz with a 50% duty cycle, and the input voltage was 8 V. The OMF signal was set to 5 Hz with a 50% duty cycle, the input voltage was 23 V, and the OMF strength was 15 mT, measured using a multi-axis teslameter (F71, Lakeshore, Westerville, OH, USA). Temperatures were measured using thermocouples and data acquisition units, with temperature recordings taken at 30 s intervals. A thermocouple wire (TT-T-40-SLE, Omega Engineering, Inc., Stamford, CT, USA) was used to collect real-time temperature data at the center of the sample surface during the experiment to determine if the sample was successfully supercooled. The supercooling chamber, placed in a 7.1 cu. ft chest freezer, had its temperature controlled by a temperature controller (Inkbird ITC-308, INKBIRD Tech. CL, Shenzhen, China; ±0.3 °C).

### 2.3. Color Measurement

The color of the salmon samples was evaluated using a hand-held colorimeter (PCE-CSM 4, PCE Americas Inc., Jupiter, FL, USA) with an aperture of 45° and 20 mm. Eight measurements were taken at random locations on each sample surface. For each condition, the top loin of the samples was trimmed, and the color was measured immediately; this was considered day 0 of storage. After 10 days of storage under each condition, the color of the sample surface was measured again. The standard deviation was taken as the error value. For comparative analysis, the L* (lightness), a* (red–green), b* (yellow–blue), and total color difference (Δ*E*) of the samples were compared. The following formula was used to calculate the total color difference (Δ*E*):

(1) $$∆E=\sqrt{{\left(L_{2}^{*}-L_{1}^{*}\right)}^{2}+{\left(a_{2}^{*}-a_{1}^{*}\right)}^{2}+{\left(b_{2}^{*}-b_{1}^{*}\right)}^{2}}$$

### 2.4. Drip Loss

The salmon samples were weighed and their weights recorded immediately after the initial sample preparation. After storage, excess drips were removed using paper towels. The final weights of the frozen samples were recorded after thawing at 4 °C for 24 h. The samples were weighed again after each treatment as final weight, and the drip loss was estimated using the following formula:

(2) $$Drip\;loss(\%)=\frac{initial\;weight-final\;weight}{initial\;weight}\times 100\;\;\;$$

### 2.5. Texture Analysis

Texture measurements were conducted using a texture analyzer (TA-TX plus, Stable Micro Systems, Godalming, UK). The textures of the refrigerated, supercooled, and frozen–thawed salmon samples were assessed individually based on a previously described method with minor modifications [20]. The samples were cut into cubic shapes (3 × 3 × 3 cm) and stored in a refrigerator. The temperature of the samples was monitored using a thermometer and T-type thermocouple wire (TT-T-40-SLE, Omega Engineering, Inc., Stamford, CT, USA), and texture analysis was performed when the temperature of the samples reached 4 °C. The texture was expressed as the change in shear force (N) after storage.

### 2.6. Thiobarbituric Acid Reactive Substances (TBARS) Test

To assess the impact of an extended supercooling state on the oxidative stability of the salmon samples, the thiobarbituric acid reactive substances (TBARS) values of the supercooled salmon samples after 10 days were compared with the TBARS values of the refrigerated (4 °C) and frozen (−18 °C) samples. Malondialdehyde (MDA) was quantified using a modified method described in reference [21]. The analysis was performed with a Genesys 20 spectrophotometer (Thermo Scientific GENESYS20, Thermo Fisher Scientific, Inc., Rochester, NY, USA). Initially, 1 g of the sample was blended with 10 mL of distilled water, homogenized, and then filtered. Then, 1 mL of the homogenized supernatant was mixed with 2 mL of 15% trichloroacetic acid (TCA) and 0.375% thiobarbituric acid (TBA) solution, along with 3 mL of 2% butylated hydroxy-toluene (BHT) (*w*/*v*) in 95% ethanol solution. All chemicals, including concentrated hydrochloric acid, TCA, TBA, and BHT, were obtained from VWR (Radnor, PA, USA). The mixture was vortexed, heated in a 90 °C water bath for 15 min, cooled to room temperature, and then centrifuged at 4000× *g* for 15 min. The absorbance of the resulting solution was measured at 532 nm. The MDA concentration was determined using a molar extinction coefficient of 1.56 × 105 M/cm and reported in mg MDA per kilogram of the sample.

### 2.7. Microbial Counts

Microbial analyses were performed at multiple time points for the different storage conditions during the storage period. Specifically, the aerobic plate count (APC) was measured on days 0, 3, 7, and 10 for the refrigerated and frozen–thawed samples. For the supercooled samples, APC values were measured on days 5 and 10. The APC was determined using the standard method outlined in the FDA’s *Bacteriological Analytical Manual* [22]. Twenty-five grams of each sample was placed into a stomacher bag containing 225 mL of sterile potassium phosphate buffer and homogenized at 250 rpm for 10 min. The resulting homogenate was then serially diluted, and 1 mL of each dilution was spread onto the surface of plate count agar. The plates were incubated at 37 °C for 48 h. Microbial counts were averaged from two plates and expressed as log CFU/g. All microbiological analysis procedures, including sample preparation, were conducted under sterile conditions.

### 2.8. Statistical Analysis

Experimental data were averaged from 3 independent experiments and analyzed using OriginPro 2021b (OriginLab, Northampton, MA, USA). A one-way ANOVA and Tukey’s test as mean comparison were performed on the variables. The experimental data showed standard deviation. Significant differences between treatments were determined at the 95% confidence level.

## 3. Results and Discussion

### 3.1. Effects of OMF and PEF Treatment on Salmon

**Figure 2:**
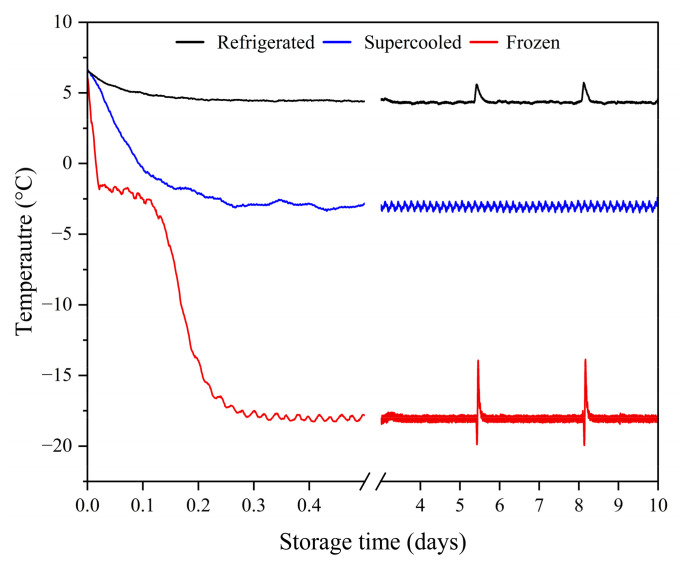
Temperature plots of refrigerated, frozen, and supercooled salmon fillets.

Figure 2 illustrates the time–temperature distribution of the salmon samples stored under freezing, refrigerated, and supercooled conditions with a combined OMF and PEF treatment. Ice formation during freezing can be identified by measuring the sample temperature. Supercooling refers to the phenomenon where the temperature of a solution or food drops below its equilibrium freezing point without ice crystallization [13]. Existing studies have shown that a PEF can inhibit ice nucleation by vibrating and displacing water molecules, which are polarized and redirected by the force momentum exerted on the dipole moment of water molecules when an external PEF acts on food samples [23]. In addition, an OMF will enhance the transition dipole moment and vibrational force of water molecules and affect the diamagnetic properties of water molecules [12]. The application of a magnetic field can weaken the van der Waals bonds between water molecules and enhance the H-O bond. This intensity means that the size of the water clusters can be controlled by using an external magnetic field [24]. The observable temperature increase during freezing is the result of latent heat released during ice formation [25]; due to the latent heat released by the freezing process, it can be observed in the time–temperature distribution that the temperature rises after a period. No latent heat release was detected in the supercooled samples during the experiment, which proved that the samples stored in the supercooling storage by the combined treatment of an OMF and PEF did not freeze, indicating that the combined treatment of an OMF and PEF effectively inhibited the nucleation of ice crystals in the samples. The freezing temperature of the tested salmon was about −2 °C. However, the temperature of the supercooled samples was reduced to −3 °C in the experiment, indicating that in the case of a combined PEF, the application of an OMF can provide stable supercooling for the experimental samples. With support, salmon samples can be stored at −3 °C for 10 days.

### 3.2. Color Change Analysis

The color of fish can be affected by many factors, including diet, environment, treatment with color fixatives such as carbon monoxide, and other packaging processes, so color itself is not an indicator of freshness. However, fish spoilage also affects color, so this experiment included color change as an indicator [26]. Table 1 shows the color of salmon fillets after 10 days of storage with the combination of OMF and PEF supercooling treatment. The L* value, representing lightness, tends to increase during storage due to changes in the fish tissue’s reflective properties due to lipid and protein oxidation. As shown in Table 1, the L* value for the refrigerated samples increased from 44.39 ± 2.39 on day 0 to 49.68 ± 1.45 on day 10, indicating a significant lightening of the flesh. For the frozen samples, the L* value showed a slight decrease, while the supercooled samples exhibited a small increase, suggesting that supercooling can maintain the lightness of a salmon fillet. The a* value, indicating the red–green spectrum, typically decreases as carotenoid pigments like astaxanthin degrade during storage. The a* value increased from 12.70 ± 0.62 to 14.34 ± 1.49 for the refrigerated samples, likely due to pigment oxidation. The frozen samples showed a decrease in the a* value, while that of the supercooled samples slightly increased, indicating a better preservation of the red color in supercooled conditions. The b* value, which measures the yellow–blue spectrum, can fluctuate due to oxidative reactions affecting the pigments. The b* value for the refrigerated samples slightly decreased. The significant differences in Δ*E* values between the refrigerated, frozen, and supercooled samples underscore the efficacy of supercooling in preserving the color quality of salmon. The lower Δ*E* values in the supercooled samples (1.96 ± 1.93) indicate that this method effectively mitigates the factors leading to pigment oxidation and structural degradation. This preservation of color not only enhances the visual appeal of the product but may also be indicative of better overall quality and freshness. However, given the 10-day storage limit of this experiment, significant changes in color parameters (L*, a*, b*) were not observed across the various storage conditions. It is important to acknowledge that the relatively short storage duration may have restricted the extent of detectable color alterations. Furthermore, the color of salmon fillets may exhibit reduced susceptibility to change due to artificial pigmentation treatments frequently employed in aquaculture to enhance the visual appeal of the flesh. These treatments can lead to more stable color characteristics over short storage periods, potentially obscuring some changes typically associated with spoilage and oxidation. Previous studies have documented that artificial pigmentation in salmon aquaculture contributes to the stability of color during storage [27,28].

### 3.3. Drip Loss

**Figure 3:**
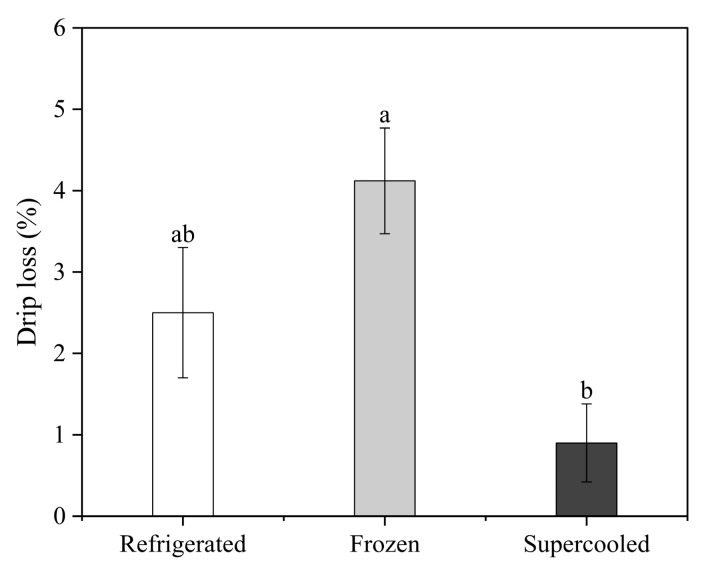
Mean values of drip loss in samples after 10 days of storage under refrigerated, frozen, and supercooled conditions. Different letters indicate significant differences within the same storage time (*p* < 0.05).

Figure 3 shows that the drip loss from the supercooled salmon samples was evaluated and compared with the refrigerated and frozen samples. The drip loss from the frozen samples was significantly higher than the refrigerated and supercooled samples (*p* < 0.05). Specifically, the drip loss values measured in each condition were 2.5 ± 0.8%, 4.12 ± 0.65%, and 0.91 ± 0.48% on day 10 for the refrigerated, frozen, and supercooled samples, respectively. According to existing studies, substantial fluid loss in frozen–thawed samples is due to cell wall rupture and protein denaturation. The drip loss in frozen–thawed salmon samples can be explained by protein denaturation and aggregation, which reduce the water-holding capacity of muscle myofibrillar proteins [29]. When meat is frozen, extracellular water freezes first, altering the water balance. As freezing progresses, existing ice crystals grow at the expense of water in the inter-fiber space, forming ice nuclei. The frozen water melts when the meat is thawed and must re-establish equilibrium with muscle proteins and salts [30]. However, ice formation squeezes, twists, and destroys the fibers and cells, resulting in less water reabsorption than usual. Consequently, the meat exhibits a reduced water reabsorption capacity compared to its pre-frozen state, and the water that has not been reabsorbed extrudes out as droplets, resulting in drip loss [31]. In contrast, the drip loss in the supercooled samples was significantly less than that of the refrigerated and frozen samples. This indicates that the OMF and PEF treatments inhibited the formation of ice nuclei during the entire storage process, preventing quality degradation. By preventing the formation of ice crystals, these treatments reduce mechanical damage to cell membranes and structural proteins that typically occurs during freezing and thawing. This results in less fluid loss during storage and subsequent thawing. 

### 3.4. Texture Analysis

**Figure 4:**
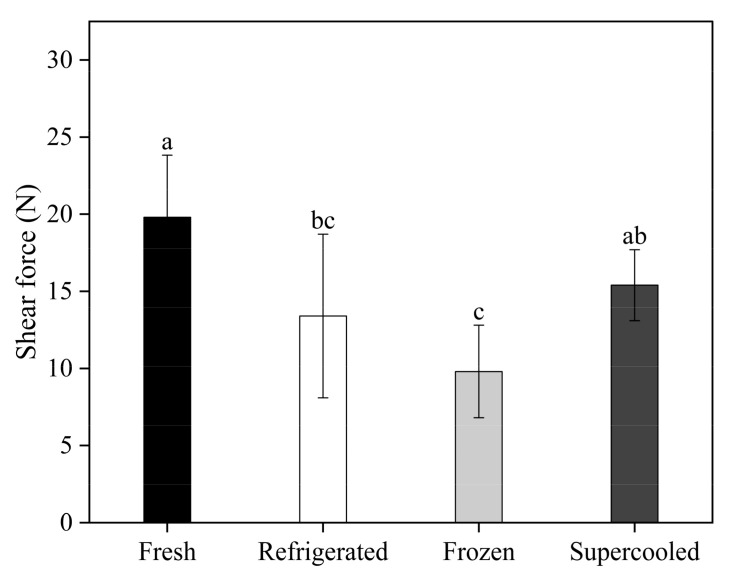
Mean values of Warner–Bratzler shear force of samples after 10 days of storage under refrigerated, frozen, and supercooled conditions. Different letters indicate significant differences according to Tukey’s test (*p* < 0.05).

The texture characteristics of salmon were evaluated, with shear force (N) as the main criterion. The shear force of the fresh salmon fillets used in this study was 19.81 ± 4.02 N. After 10 days of storage, the Warner–Bratzler shear force (WBSF) values of samples were 13.44 ± 5.37 N, 9.82 ± 3.18 N, and 15.49 ± 2.32 N for the refrigerated, frozen, and supercooled conditions, respectively (Figure 4). The shear force of the supercooled and refrigerated samples was significantly different from that of the fresh samples (*p* < 0.05). Although the WBSF of the supercooled samples was significantly lower than that of the fresh samples (*p* < 0.05), the value for the supercooled salmon was significantly higher than those of the frozen and refrigerated samples after 10 days of storage (*p* < 0.05). Previous studies have indicated that the decrease in hardness during storage is linked to drip loss and the breakdown of structural proteins by proteolytic enzymes [32]. Additionally, texture changes in salmon during storage are influenced by the density of muscle fibers, various intrinsic biological factors, such as collagen and fat content, and autolytic and microbial processes that occur post-mortem [33]. The experimental results demonstrate that the combined application of OMF and PEF supercooling effectively prevents the formation of ice nuclei during storage, thereby maintaining the hardness of the samples.

### 3.5. Thiobarbituric Acid Reactive Substances (TBARS) Test

**Figure 5:**
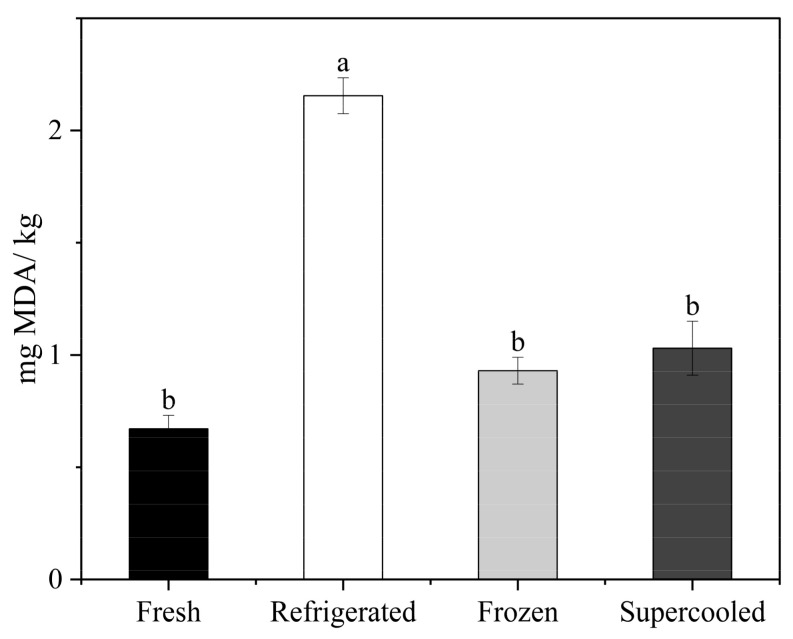
Mean values of degree of lipid oxidation of samples after 10 days of storage under refrigerated, frozen, and supercooled conditions. Different letters indicate significant differences according to Tukey’s test (*p* < 0.05).

Lipid oxidation is a complex chemical process involving many factors, such as oxygen, heat, microorganisms, and enzymes [34]. TBARS values are used to reflect the degree of lipid oxidation, which, in the case of salmon, can lead to the formation of rancid odors, indicating a decrease in food quality and potentially leading to food poisoning if consumed [35]. Additionally, the TBARS values of salmon are affected by storage temperature. Salmon contains unsaturated fatty acids, such as docosahexaenoic acid (DHA), which are susceptible to oxidative species, forming hydroperoxides that spoil secondary products such as malondialdehyde (MDA) [36]. As shown in Figure 5, after 10 days of storage, the TBARS values in all groups increased significantly (*p* < 0.05), with the refrigerated samples exhibiting significantly higher values than the other two treatments (*p* < 0.05). These results indicate that the lipid oxidation of salmon fillets is promoted under temperature fluctuations during supercooling storage. The greater the degree of temperature fluctuation, the more significant the increase in TBARS values. For samples stored for 10 days, the MDA content of the refrigerated samples was much higher than that of the other samples. The MDA content of the supercooled samples was slightly higher than in the frozen–thawed samples, though the difference was not significant. However, all of the treated samples had a higher MDA content than the fresh samples. Therefore, preserving fish by supercooling is more effective in preventing lipid oxidation than cold storage. Supercooling with an OMF and PEF maintains a stable subzero temperature without ice crystal formation. This stability reduces the rate of lipid oxidation, as temperature variations can accelerate the formation of free radicals and oxidative species [17]. Additionally, the application of an OMF and PEF can inhibit the activities of enzymes that catalyze lipid oxidation. Enzymes such as lipoxygenase are less active at stable subzero temperatures, leading to a reduction in the formation of primary and secondary oxidation products [18].

### 3.6. Microbiological Analysis

**Figure 6:**
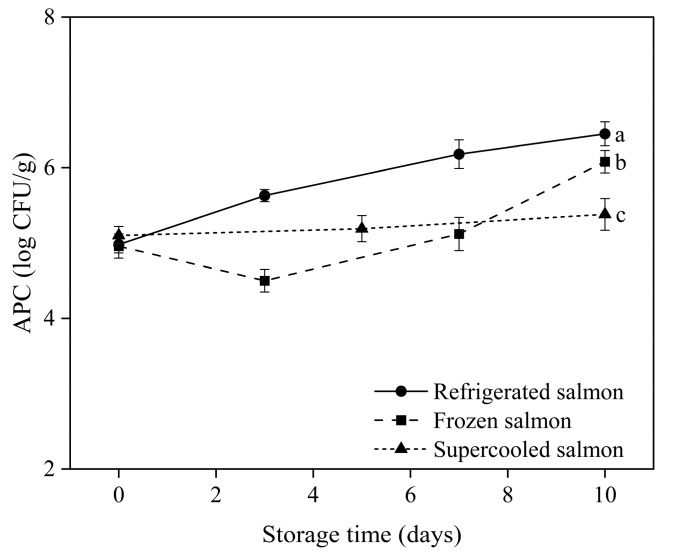
Mean value of APC during 10 days of storage under refrigerated, frozen, and supercooled conditions. Different letters indicate significant differences according to Tukey’s test (*p* < 0.05).

The aerobic plate count (APC) test was performed on salmon stored under three distinct conditions: refrigeration, freezing, and supercooling. Figure 6 illustrates that the APC of refrigerated and frozen–thawed salmon exhibited significant growth over the 10-day storage period. Conversely, the APC values of the supercooled salmon samples remained relatively stable during the same period. According to Kim et al. [37], the microbiological quality of raw ready-to-eat seafood can be categorized into three levels: satisfactory (APC < 5 log CFU/g), acceptable (5 log CFU/g ≤ APC ≤ 6 log CFU/g), and unsatisfactory (APC ≥ 6 log CFU/g). The refrigerated samples reached ‘unsatisfactory’ APC levels of 6.18 ± 0.16 log CFU/g after 7 days, while the frozen samples showed 6.08 ± 0.15 log CFU/g after 10 days. In contrast, the supercooled salmon maintained an average APC of 5.38 ± 0.21 log CFU/g even after 10 days, which falls within the ‘acceptable’ range. The supercooled salmon samples were able to sustain a low subzero temperature, effectively slowing down bacterial growth during storage. Additionally, these samples did not require a thawing step before consumption as the fish did not freeze. While low-temperature thawing processes are beneficial in minimizing physical and biochemical changes in meat and fish products, the conventional thawing method used in this study could potentially increase bacterial populations [38,39]. 

In addition to the aerobic bacteria group, it is important to consider other significant spoilage bacteria such as *Pseudomonas* spp., *Enterobacteriaceae*, and H_2_S-producing bacteria. These microorganisms are well-known spoilage agents in seafood. *Pseudomonas* spp. are psychrotrophic bacteria that can grow at refrigeration temperatures, leading to off-odors and slime production. *Enterobacteriaceae* includes coliforms and other bacteria that can indicate hygiene issues and potential pathogenic contamination. H_2_S-producing bacteria like Shewanella putrefaciens contribute to spoilage by producing hydrogen sulfide, which causes discoloration and off-odors [40]. Studies on superchilling storage, which is similar to supercooling, have demonstrated its effectiveness in inhibiting the growth of spoilage microorganisms. Superchilling involves storing food at temperatures just below the initial freezing point, maintaining product quality while reducing microbial growth. Sørensen et al. (2020) [41] showed that superchilling combined with modified atmosphere packaging effectively limited microbial growth in Atlantic cod, leading to extended shelf life and improved microbial quality. Similarly, Bono et al. (2017) [42] reported that superchilling storage significantly reduced the growth of spoilage bacteria in pelagic fish species.

## 4. Conclusions

This experiment aimed to study the effects of supercooling storage on the quality of farm-raised salmon fillets. Using OMF and PEF treatments to inhibit ice nucleation, salmon fillets were stored at −3 °C for 10 days in a supercooled state. The supercooled sample indicated a reduction in total color change (Δ*E*) and showed significantly lower drip loss and minimized texture degradation compared to the refrigerated and frozen samples. Moreover, supercooling effectively minimized microbial growth assessed by APC methods and MDA production for 10 days of preservation. These results demonstrate that supercooling effectively preserves the quality of perishable seafood, offering promising potential to conventional refrigeration and freezing methods.

## Figures and Tables

**Table 1:** Color parameters of salmon fillets under different storage conditions.

Treatment	Storage Days	L*	a*	b*	Δ*E*
Refrigerated	0	44.39 ± 2.39 ^a^	12.70 ± 0.62 ^a^	12.50 ± 1.17 ^a^	
	10	49.68 ± 1.45 ^b^	14.34 ± 1.49 ^b^	12.24 ± 1.34 ^a^	5.81 ± 1.46 ^a^
Frozen	0	46.88 ± 1.83 ^a^	13.92 ± 1.35 ^a^	15.02 ± 1.95 ^a^	
	10	44.76 ± 2.01 ^b^	13.16 ± 0.95 ^b^	14.78 ± 1.16 ^a^	4.21 ± 1.74 ^b^
Supercooled	0	51.24 ± 4.53 ^a^	12.88 ± 0.91 ^a^	14.46 ± 0.70 ^a^	
	10	53.45 ± 4.19 ^a^	13.96 ± 1.77 ^b^	15.10 ± 1.39 ^a^	1.96 ± 1.93 ^c^

Different letters in the same column indicate significant differences (*p* < 0.05).

## Data Availability

The original contributions presented in the study are included in the article, further inquiries can be directed to the corresponding authors.
